# Three doses of an inactivation-based COVID-19 vaccine induces cross-neutralizing immunity against the SARS CoV-2 Omicron variant

**DOI:** 10.1080/22221751.2022.2044271

**Published:** 2022-03-03

**Authors:** Xiaoling Yu, Xiangrong Qi, Yu Cao, Peiyao Li, Li Lu, Pingping Wang, Yuchen Feng, Jie Yang, Huihui Wei, Lixian Guo, Mingyue Sun, Qiang Liu, Jing Lv, Yingmei Feng

**Affiliations:** a Gobond Testing Technology (Beijing) Co., Ltd., Beijing, People’s Republic of China; b Department of Science and Development, Beijing Youan Hospital, Capital Medical University, Beijing, People’s Republic of China; c Yearth Biotechnology Co. Ltd., Changsha, People’s Republic of China

**Keywords:** Omicron variant, SARS-CoV-2, Vaccine, Neutralizing antibody, Pseudotyped virus

## Abstract

The immunity potency upon natural infection or vaccination is the main concern for the vaccine strategy of severe acute respiratory syndrome coronavirus 2 (SARS COV-2 variant), especially the recently reported Omicron variant (B.1.1.529). In this study, 200 recipients immunized with three doses of a COVID-19-inactivated vaccine were enrolled, whose serum samples were collected within 2 months after the third immunization. The neutralizing activity of sera against the pseudotyped Omicron variant, prototype, and Delta variant was determined. Our results demonstrated that the positive neutralization activity was 95.5% for the Omicron variant, 99.5% for the prototype, and 98.5% for the Delta variant. The geometric mean titers (GMT) for the Omicron variant was 49 and maintained sustained immune levels for 2 months, which decreased by 4.9-fold and 3.0-fold compared with the prototype (GMT, 239) and Delta variant (GMT, 148), respectively. In summary, our study demonstrated that three doses of a COVID-19-inactivated vaccine effectively yielded potent cross-neutralizing activity against the Omicron variant at 2 months after the third vaccination.

## Introduction

On November 25, 2021, a new SARS COV-2 variant (Omicron) was reported in South Africa, and it was designated as the fifth variant of concern by the World Health Organization [1]. Early epidemiological evidence has shown that Omicron is spreading rapidly, especially in young people, and has replaced Delta as the predominant variant worldwide. As of December 15, 2021, the Omicron variant is already present in 80 countries, including China [2]. The Omicron variant has more than 30 mutations in the Spike protein with 15 mutations located in the receptor-binding domain, the binding site of ACE-2 receptor-mediated virus–cell interaction [3]. Whether the mutations will cause the Omicron variant to be more infectious or result in more serious symptoms than other variants is unclear and whether it can escape immunity induced by natural infection or vaccination has become the largest concern. A study has shown significant immune escape of the Omicron variant in COVID-19 convalescent patients infected with the original SARS COV-2 strain [4].

A booster vaccination has been approved for recipients that have received a two-dose vaccine regime more than 6 months previously, that is very effective at inducing high neutralizing antibody titres against other variants [5]. However, it is unclear whether the immunity elicited by three doses of an inactivated vaccine is maintained for the Omicron variant with high mutations.

In this study, we constructed a pseudotyped SARS-CoV-2 Omicron variant and detected serum neutralizing antibodies in 200 young people immunized with three doses of an inactivation-based vaccine.

## Methods

Serum samples from 200 COVID-19 vaccinees were collected within 2 months after the third immunization at the Beijing YouAn Hospital on December 10, 2021. All participants (54 males and 146 females) had received three doses of a homologous inactivation-based vaccine. Among them, 122 participants had accepted vaccine A and 78 participants had received vaccine B (Supplementary Table 1). Informed consent forms were signed and ethical approval was obtained from Beijing YouAn Hospital, Capital Medical University (#LL-2021-159-K).

A pseudovirus strain of SARS-Cov-2 Omicron was constructed with 32 mutations in the S protein. Pseudoviruses of the prototype and Delta variant were also constructed (Supplementary Table 2).

The SARS-CoV-2 pseudovirus was prepared by transfecting 293T cells with S protein expression plasmids. G*ΔG-VSV *(Kerafast, Boston, MA)* was added by providing genomes of VSV. After collection and filtration, the pseudotyped viruses were titrated in Vero cells [6]. A serum pseudovirus neutralization test was conducted by a chemiluminescence method. The 50% neutralization dilution (ND50) level was calculated using the Reed-Muench method [6]. Data were plotted using GraphPad Prism 8 (GraphPad, San Diego, CA). A t-test and unpaired t-test with Welch’s correction were used for statistical analysis. Significance was considered at *P *< 0.05.

## Results

A total of 200 volunteers were recruited, which included 54 males and 146 females. They were aged 26.1 ± 5.8 years old (Supplementary Table 1). In the study, a pseudovirus strain of SARS-Cov-2 Omicron was constructed, with 32 mutations within S protein. At the same time, the pseudoviruses of Wuhan-1 strain and Delta variant were also constructed (Supplementary Table 2).

**Figure 1.**
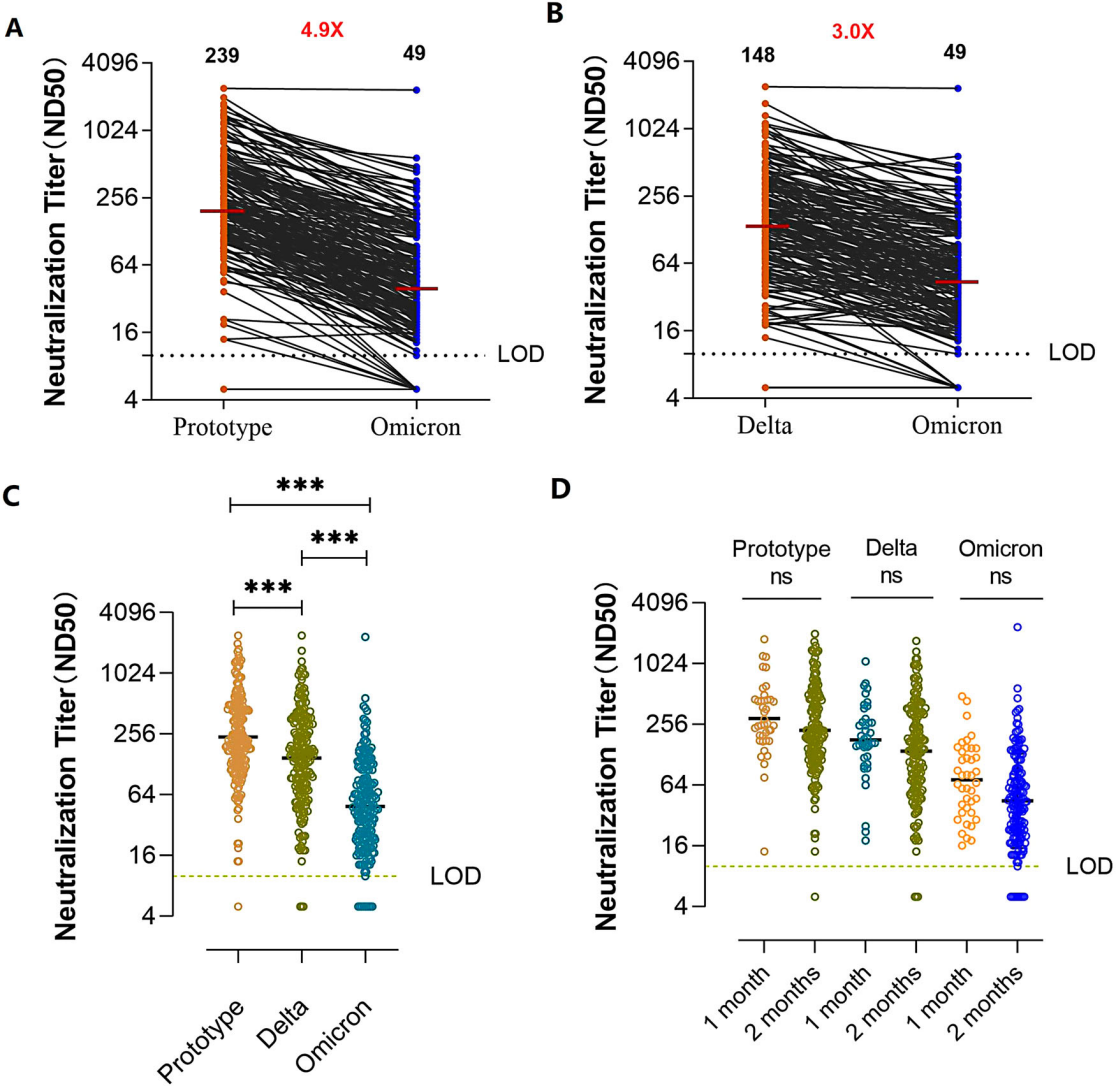
Neutralizing antibody production following booster vaccination. (A, B) Neutralization titres against the prototype, Delta, and Omicron SARS Cov-2 variants in individuals after the third vaccination within 2 months. The limitation of detection (LOD) was *1:10.* Titres below the LOD were set to 0.5 times the LOD. Numbers in black above each virus strain are geometric mean titers (GMT) of the neutralization titers (ND50) resulting in serum pseudovirus neutralization test. Numbers in red denote fold-change in GMT between different pseudovirus. (C) Comparison of neutralization titres against the prototype, Delta, and Omicron SARS Cov-2 variants in individuals after the third vaccination within 2 months (*n* = 200, *P*-values were obtained by the unpaired t-test, ***P < 0.0001). (D) Neutralization activity of sera from individuals at various time points after booster immunization against different SARS Cov-2 variants. Prototype, 1 month vs 2 months: *P *= 0.4698, Delta variant, 1 month vs 2 months: *P *= 0.97, Omicron variant, 1 month vs 2 months: *P *= 0.46; ns, not significant.

The neutralization-positive rates of serum against the pseudovirus strain of Omicron, prototype, and Delta variant were 95.5%, 99.5%, and 98.5% respectively (Table 1). The geometric mean titers (GMT) of the serum pseudovirus neutralization test for Omicron variant was 49, which was decreased by 4.9-fold compared with the prototype strain (GMT, 239) (Figure 1(A)). Compared with the Delta variant (GMT, 148), the GMT for Omicron variant was decreased by 3.0-fold (Figure 1(B)). The overall comparison of neutralizing titres against each variant is shown in Figure 1(C). There was no significant difference in the GMTs for the Omicron pseudovirus between serum collected within 1 and 2 months after the third immunization, which implied maintenance of the cross-neutralizing activity for the Omicron variant at 2 months after the three-dose immunization. This result was consistent with that of the prototype and Delta variant (Figure 1(D)). The neutralization activities against Omicron in serum from participants that had received vaccine A were similar to those of participants that had received vaccine B (Supplementary Figure 1).

**Table 1.** Neutralization-positive rates against the pseudotyped SARSCov-2 variant.

	Prototype	Delta	Omicron
**Positive rate**	99.50%	98.50%	95.50%
**Negative rate**	0.50%	1.50%	4.50%

Note: Titres up *1:10* were considered to be positive.

## Discussion

The Omicron variant, which harbours substantially more mutations than prior variants, may escape humoral immunity induced by natural infection or primary vaccination. A retrospective analysis of routine epidemiological surveillance data has suggested that the Omicron variant is associated with an increase in the risk of reinfection after a primary infection [7], which indicates that the Omicron variant has the ability to evade immunity induced by prior infection. Recent studies have shown a significant reduction in the neutralization sensitivity in serum of patients who recovered from previous COVID-19 infections [4]. The neutralizing activity against Omicron variant after two-dose vaccination showed a 10–25-fold reduction [8,9].

In this study, we mainly evaluated the neutralization activity of vaccinated sera against Omicron in a large young population to determine whether three doses of an inactivated-based COVID-19 vaccine induced cross-neutralizing immunity against the SARS CoV-2 Omicron variant. Some data have indicated that a third dose of BNT162b2 increases neutralizing antibody titres against the Omicron variant by 25-fold compared with two doses. Thus, titres after the booster dose were associated with high levels of protection [8]. After the third dose of the homologous inactivated vaccine, compared with the prototype and Delta strain, the neutralizing activity against the Omicron variant was decreased (*P *< 0.05). The GMT against Omicron variant remained above the baseline, which implied cross-neutralizing protection elicited by both types of inactivated vaccine.

Neutralization responses and vaccine effectiveness decrease with increased time post-vaccination. All serum samples in this study were collected within 2 months after booster vaccination. The long-term effectiveness of booster vaccination needs to be evaluated. This study used an in vitro assay to test neutralization activity on the basis of a pseudotyped virus system, which is widely used to evaluate the humoral immune status elicited by natural infection or a vaccine. More clinical studies need to be carried out to evaluate the immune protection efficacy against the Omicron variant.

Taken together, we demonstrated that the Omicron variant escapes vaccine-induced immunity compared with the prototype and Delta variant. Receiving three doses of an inactivated vaccine effectively yields potent cross-neutralizing activity against the Omicron variant.

## Supplementary Material

Supplemental Material

Supplemental Material
