# Supplementary figures and images for: Three doses of an inactivation-based COVID-19 vaccine induces cross-neutralizing immunity against the SARS CoV-2 Omicron variant
Source: Emerg Microbes Infect. 2022 Mar 3;11(1):749–52. doi: 10.1080/22221751.2022.2044271 (PMC8903785; doi:10.1080/22221751.2022.2044271)

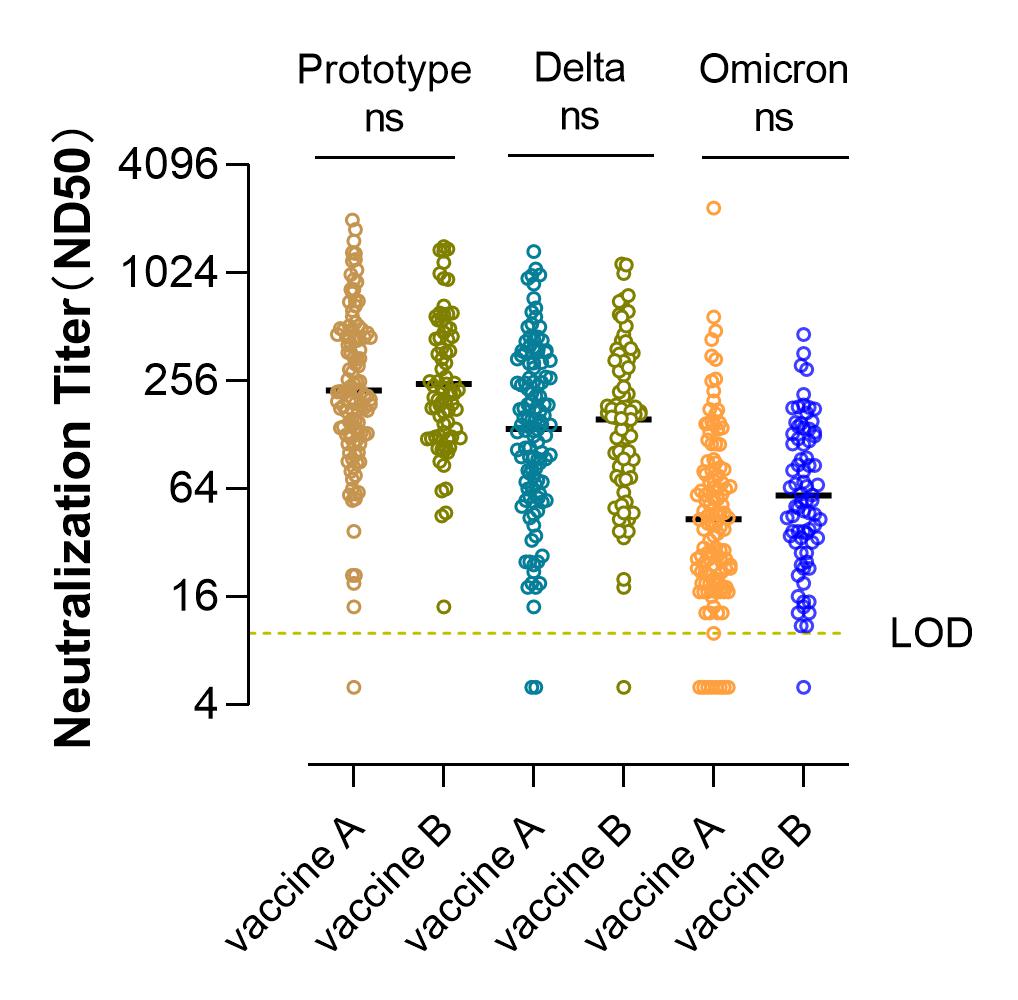

Supplement: Supplemental Material [file TEMI_A_2044271_SM4051.jpg]
