# Supplementary material for: Three doses of an inactivation-based COVID-19 vaccine induces cross-neutralizing immunity against the SARS CoV-2 Omicron variant
Source: Emerg Microbes Infect. 2022 Mar 3;11(1):749–52. doi: 10.1080/22221751.2022.2044271 (PMC8903785; doi:10.1080/22221751.2022.2044271)
Supplement: Supplemental Material [file TEMI_A_2044271_SM4002.docx]

**Supplemental materials**

**Three doses of an inactivation-based COVID-19 vaccine induces cross-neutralizing immunity against the SARS CoV-2 Omicron variant**

Xiaoling Yu^1^*, Xiangrong Qi^1^*, Yu Cao*^2^, Peiyao Li^1^*, Li Lu^3^, Pingping Wang^1^, Yuchen Feng^1^, Jie Yang^1^, Huihui Wei^1^, Lixian Guo^1^, Mingyue Sun^1^, Qiang Liu^1#^, Jing Lv^1#^, Yingmei Feng^2#^

1. Gobond Testing Technology (Beijing) Co., Ltd., 38 Yongda Road, Beijing 102629, China
2. Department of Science and Development, Beijing Youan hospital, Capital Medical University, Beijing 100069, China
3. Yearth Biotechnology Co. Ltd., Changsha, Hunan 410205, China

*These authors contributed equally to this work.

^#^Correspondence:

Yingmei Feng MD, PhD

Email: [yingmeif13@sina.com](mailto:yingmeif13@sina.com); yingmeif13@ccde.edu.cn

Jing Lv MD, PhD

Email: [lvjing@gobondtest.com](mailto:lvjing@gobondtest.com)

Qiang Liu PhD

Email: [liuqiang@gobondtest.co](mailto:liuqiang@gobondtest.co)m

**Table 1. Basis information of participants recruited in the study**

| **Gender** | | **Age** | | **Time post immunization** | | **Vaccine type** | |
| --- | --- | --- | --- | --- | --- | --- | --- |
| Male | Female | 19-25 years | 26-40 years | <1month | 1-2 months | Vaccine A | Vaccine B |
| 53 | 146 | 109 | 90 | 40 | 159 | 122 | 78 |

**Table 2.** **The mutation sites of S genes of different pseudotypes of SARS Cov-2 variants**

| **Pseudotyped virus** | **Pangolin**  **lineage** | **Mutant site in S gene**  **（Genbank: MN908947as reference sequence）** |
| --- | --- | --- |
| **Prototype** | **/** | / |
| **Delta** | *B.1.617.2* | *T19R, G142D, E156del, F157del, R158G, L452R, T478K, D614G, P681R, D950N* |
| **Omicron** | *B.1.1.529* | *A67V, H69del, V70del, T95I, G142D, V143del, Y144del, Y145del, N211del, L212I, ins214EPE, G339D, S371L, S373P, S375F, K417N, N440K, G446S, S477N, T478K, E484A, Q493R, G496S, Q498R, N501Y, Y505H, T547K, D614G, H655Y, N679K, P681H, N764K, D796Y, N856K, Q954H, N969K, L981F* |


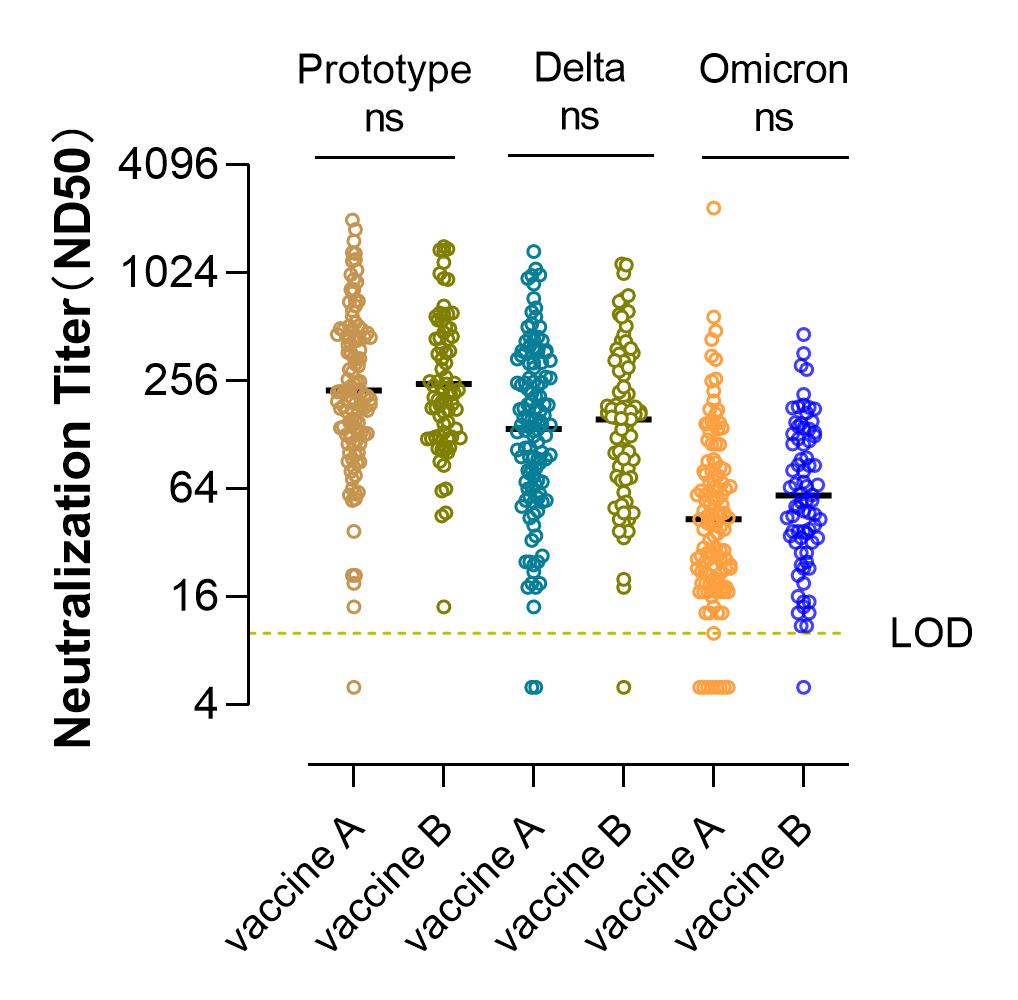


**Supplementary Figure 1: The sVNT titer against the prototype, Delta variant and Omicron variant in inactivated-based vaccine A group and inactivated-based vaccine B group.** GMTs were used to compare the neuralization activities of serum between different pseudotyped viruses. Prototype, vaccine A vs vaccine B: *P*=0.9722, Delta variant, vaccine A vs vaccine B: *P*=0.6296, Omicron variant, vaccine A vs vaccine B: *P*=0.8823, ns, not significant.
